# Propofol infusion in critically ill patients is not associated with mitochondrial dysfunction or altered serum interleukin levels in the early sepsis response

**DOI:** 10.62675/2965-2774.20260273

**Published:** 2026-03-03

**Authors:** Wagner Luis Nedel, Luis Valmor Portela

**Affiliations:** 1 Grupo Hospitalar Conceição Intensive Care Unit Porto Alegre RS Brazil Intensive Care Unit, Grupo Hospitalar Conceição - Porto Alegre (RS), Brazil.; 2 Universidade Federal do Rio Grande do Sul Instituto de Ciências Básicas da Saúde Neurotrauma and Biomakers Laboratory Porto Alegre RS Brazil Neurotrauma and Biomakers Laboratory, Instituto de Ciências Básicas da Saúde, Universidade Federal do Rio Grande do Sul - Porto Alegre (RS), Brazil.

## INTRODUCTION

The immune status of patients with sepsis changes dynamically across different stages, involving complex interactions among multiple immune cells and molecular mechanisms. A dysregulated balance between pro-inflammatory and anti-inflammatory responses can generate sepsis-induced immunosuppression.^(1)^ A persistent immunosuppressive state is characterized by immune cell exhaustion, particularly in mononuclear cells, whereas late-stage mortality is frequently associated with secondary infections.^(2)^ Immune cell exhaustion is associated with increased production of reactive oxygen species and compromised mitochondrial respiration, which is representative of septic mononuclear cells.^(3)^ Although not yet fully characterized, environmental factors related to sepsis management, such as oxygen levels, nutrient availability, pharmacological interventions, and even inflammatory effector profiles, may significantly influence whether immune cells progress toward exhaustion or recover and return to homeostasis.

Critically ill patients, especially those with sepsis, require various supportive therapies throughout the course of the disease, including the use of antimicrobials, vasopressors, and sedatives, particularly in patients undergoing invasive mechanical ventilation. Currently, there is growing interest in the potential role of adjunct therapies as enhancers of an immunosuppressive state in sepsis.^(4,5)^ Propofol has been reported to increase mitochondrial reactive oxygen species production and impair mitochondrial metabolism in animal and cellular models.^(6)^ Interestingly, in the perioperative setting, propofol is associated with higher anti-inflammatory cytokine levels,^(7)^ and in animal models of acute kidney injury, propofol attenuates oxidative renal damage,^(8)^ suggesting potential organ-protective and immunomodulatory properties beyond its anesthetic effects.

Given the growing interest in this topic in the current literature and the release of new data evaluating the potential role of supportive treatments in modulating the immune response in patients with sepsis, we evaluated the association between propofol use and mitochondrial and inflammatory variables in the early sepsis response.

## METHODS

We conducted a post hoc analysis of a cohort study that prospectively evaluated consecutive patients admitted to four intensive care units (ICUs) at a tertiary academic hospital. The original cohort has been described previously.^(9)^ This study was approved by the local Ethics Committee (Plataforma Brazil number 66240017.0.0000.5530). Adult patients (> 18 years of age) admitted to the ICU with sepsis and persistent hypotension were enrolled. Persistent hypotension was defined as the need for vasopressors to maintain a mean arterial pressure > 65mmHg after initial fluid administration. All blood samples for mitochondrial measurements were collected as described previously.^(9)^ Patients received propofol-based sedation according to the preference of their attending physician, with fentanyl-based analgesia via continuous infusion. Control patients received continuous or bolus midazolam-based sedation with fentanyl-based analgesia, continuous fentanyl infusion (analgesia-based sedation), or no sedation. These choices were made according to the physician's discretion. Mitochondrial respiration was assessed on days 1 (D1) and 3 (D3) and delta (D3 - D1) in the isolated lymphocytes. Mitochondrial variables were measured using high-resolution oxygraphy (Oxygraph-2k; Oroboros Instruments, Innsbruck, Austria) at 37°C. This protocol allows for the assessment of Complex I (CI), Complex II (CII)-linked respiration, and Biochemical Coupling Efficiency (BCE). We also measured the serum levels of IL-1, IL-6, and IL-10 at the same time points to characterize the systemic inflammatory response. IL-1, IL-6, and IL-10 levels were measured using an enzyme-linked immunosorbent assay (Invitrogen, Waltham, Massachusetts, USA) and were measured in pg/mL. The main objective of this study was to compare patients who received propofol during the first 72 h of ICU admission with those who did not, with respect to mitochondrial respiratory endpoints on D1 and D3. The secondary outcome was the association between propofol use and ILs levels on D1 and D3. We also compared the delta in mitochondrial and inflammatory variables with the cumulative propofol dose infused at the same time points.

Descriptive statistics included frequencies and percentages for categorical variables and means, standard deviations (SD), confidence intervals (CI), medians, and interquartile ranges (IQR) for continuous variables. The association between propofol use and levels of mitochondrial and inflammatory variables was analyzed using the Student's t test or the Mann-Whitney U test, depending on the distribution of the variables. The correlation between the cumulative propofol dose and absolute levels of mitochondrial and inflammatory variables was analyzed using Spearman's correlation test. We also explored the interactions among the D1 values of mitochondrial variables, propofol use, and the D3 values of these variables. To this end, we conducted an analysis using a generalized linear model, with the delta of the mitochondrial variables as the dependent variable and the mitochondrial variable on D1 and propofol use as covariates. Statistical analyses were performed using Jamovi software.

## RESULTS

Ninety patients were analyzed for mitochondrial variables on D1, 75 on D1 and D3, and 64 for inflammatory variables on D1 and D3. Patients who used propofol had similar SAPS 3 scores at ICU admission compared with those who did not use propofol. In addition, patients who used propofol had SOFA scores on D1 of sepsis similar to those who did not use propofol. The main clinical characteristics, dichotomized by propofol and non-propofol use, are presented in table 1. The 28-day mortality of the entire cohort was 46%. There was no difference in 28-day and hospital mortality between propofol users and non-users. Twenty-two patients (19%) received propofol, and the median (interquartile range) dose of propofol during this period (D1 - D3) was 1,130mg (425 - 2,400mg).

**Table 1:** Clinical variables in propofol and non-propofol users

Variable		Propofol (n = 22): mean ± SD or median (IQR) or proportion	Non-propofol (n = 68): mean ± SD or median (IQR) or proportion	p value
Age		61.7 ± 18.2	65.9 ± (15.2	0.28
Male:female ratio		11/11	39/29	0.55
SAPS 3 score		75 ± 16	75 ± 11	0.97
SOFA score at sepsis diagnosis		9 (7 - 11)	8 (6 - 10)	0.46
SOFA improvement at day 3		16/22	42/68	0.35
Clinical (*versus* surgical) admission		15/22	40/68	0.43
Sepsis foci				0.34
	Abdominal	7/22	29/68	
	Pulmonary	13/22	28/68	
	Primary bloodstream infection	1/22	6/68	
	Urinary	1/22	3/68	
Comorbidities				
	Cancer	4/22	15/68	0.70
	Cirrhosis	1/22	6/68	0.51
	COPD	3/22	9/68	0.96
	Chronic kidney disease	4/22	6/68	0.22
	Diabetes	4/22	21/68	0.25
	Hypertension	11/22	21/68	0.10
	Maximum NE dose at day 1 (µg/kg/min)	0.21 (0.09 - 0.58)	0.22 (0.12 - 0.41)	0.93
	CRP at sepsis diagnosis	150 (63 - 264)	173 (106 - 235)	0.53
Mitochondrial variables				
	Complex I respiration (pmol O_2_.^s-1^.10^-6^ cells)	265 (177 - 415)	250 (172 - 381)	0.81
	Complex II respiration (pmol O_2_.^s-1^.10^-6^ cells)	542 (399 - 758)	538 (379 - 774)	0.90
	BCE	0.295 ± 0.134	0.308 ± 0.106	0.63
	Delta Complex I respiration (pmol O_2_.^s-1^.10^-6^ cells)	144 (28.6 - 314)	108 (3.8 - 226)	0.58
	Delta Complex II respiration (pmol O_2_.^s-1^.10^-6^ cells)	192 (-49.6 - 393)	155 (-85.7 - 451)	0.87
	Delta BCE	0.03 (-0.11 - 0.12)	0.01 (-0.09 - 0.14)	0.67
Inflammatory variables at sepsis diagnosis				
	IL-1 (pg/mL)	39.8 (10.3 - 543)	34.2 (16.7 - 487)	0.63
	IL-10 (pg/mL)	170 (114 - 223)	193 (165 - 244)	0.31
	IL-6 (pg/mL)	117 (29.1 - 308)	64 (31.5 - 179)	0.51
	Delta IL-1 (pg/mL)	2.7 (-26.9 - 61.4)	-6.5 (-21.1 - 13.8)	0.22
	Delta IL-10 (pg/mL)	19.1 (-21.3 - 99)	3.3 (-37.2 - 159)	0.98
	Delta IL-6 (pg/mL)	-22.1 (-214 - 24.4)	-5 (-58.8 - 20.6)	0.61
New-onset HD		9/22	20/68	0.32
Mechanical ventilation		20/22	57/68	0.41
Reinfection during hospital stay		8/22	30/68	0.52
28-day mortality		8/22	34/68	0.27
Hospital mortality		11/22	41/68	0.40

SD - standard deviation; IQR - interquartile range; SAPS - Simplified Acute Physiology Score; SOFA - Sequential Organ Failure Assessment; COPD - chronic obstructive pulmonary disease;

NE - norepinephrine; CRP - C-reactive protein; BCE - biochemical coupling efficiency; IL - interleukin; HD - hemodialysis.

**Figure 1:**
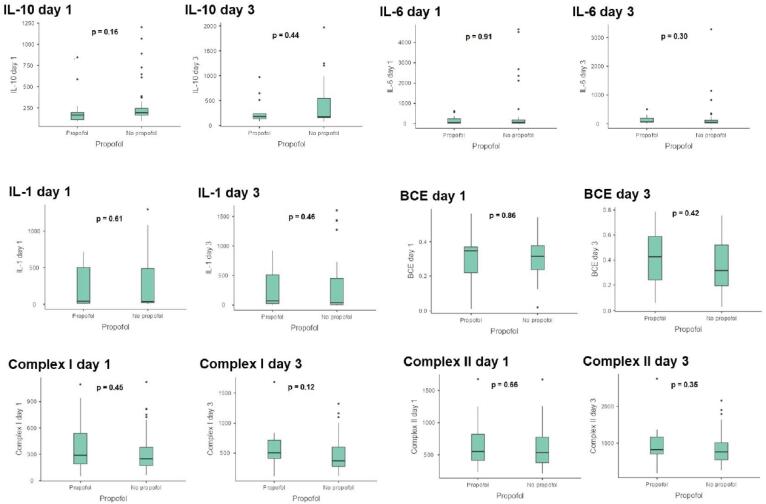
Box-and-whisker plot evaluating propofol users and non-users regarding mitochondrial and inflammatory variables.

The basal mitochondrial and inflammatory variables (day 1 measurements) did not differ between patients who used propofol and those who did not at the time of sepsis diagnosis. In addition, we did not observe any differences between propofol users and non-users regarding mitochondrial and inflammatory variables on day 3. Figure 1 shows the measured levels of variables at two time points. However, due to the low number of patients included, the statistical power for the primary outcome is low: Complex I respiration (day 1) 16%, Complex II respiration (day 1) 12%, BCE 3% (day 1), Complex I respiration (day 3) 23%, Complex II respiration (day 3) 14%, BCE (day 3) 13%, interleukin (IL) 6 (day 3) 9%, IL-1 (day 3) 4%, IL-10 (day 3) 18%. There was no interaction between the use of propofol and the delta of mitochondrial variables when stratified by propofol use and basal (D1) mitochondrial variable measurements: BCE (β coefficient −0.244, 95%CI −0.815 to 0.326; p = 0.396), Complex I respiration (β coefficient −0.29, 95%CI −0.83 to 0.25; p = 0.28), and Complex II respiration (β coefficient −0.25, 95%CI −0.78 to 0.27; p = 0.338).

IL - interleukin; BCE - biochemical coupling efficiency.

We also evaluated the association between the cumulative propofol dose between D1 and D3 and the delta values (D3 - D1) of the inflammatory and mitochondrial variables. The cumulative propofol dose was not associated with the delta of inflammatory variables. The Spearman's correlation coefficient with delta IL-10 was −0.15 (p = 0.62), delta IL-6 was 0.14 (p = 0.64), and delta IL-1 was −0.19 (p = 0.52). In addition, the cumulative propofol dose was not associated with the delta in mitochondrial variables. The Spearman's coefficient with delta Complex I respiration was −0.10 (p = 0.72), with delta Complex II respiration was −0.20 (p = 0.49), and delta BCE was −0.30 (p = 0.28).

## DISCUSSION

Propofol is a popular intravenous sedative drug commonly administered in ICU settings. It is known to have anti-inflammatory and antioxidant effects when used as an anesthetic.^(10)^ Therefore, it is relevant to explore the interaction between propofol administration and immune response mediated by cytokine levels and metabolic activity of immune cells in critically ill patients. Similar to the findings of Miller et al.,^(5)^ we did not find any association between propofol use and mitochondrial dysfunction. Despite the different methodologies used to measure mitochondrial activity, neither result indicated a potential risk with short-term use or usual doses of propofol. However, it is speculated that the use of high doses or prolonged use of propofol may lead to propofol infusion syndrome, which is characterized by extreme mitochondrial dysfunction related to drug use. This rare complication was not observed in our study, since our patients received a relatively low dose of propofol. The small sample size of our study, however, prevented us from drawing definitive conclusions regarding the absence of interactions between the variables. These findings lay important groundwork for future research with larger cohorts to confirm and expand on these observations.

## CONCLUSION

We could not determine an association between mitochondrial dysfunction in immune cells and propofol use. We did not find an association between interleukin levels commonly elevated in the acute phase of sepsis and whether propofol was used, or between the cumulative dose of propofol and the variability of interleukin levels during the same period. This lack of association, however, is merely speculative and hypothesis-generating, since our small sample size does not allow for more definitive conclusions about these interactions.

## Data Availability

After publication the data will be available on demand to authors.
